# A model and typology of collaboration between professionals in healthcare organizations

**DOI:** 10.1186/1472-6963-8-188

**Published:** 2008-09-21

**Authors:** Danielle D'Amour, Lise Goulet, Jean-François Labadie, Leticia San Martín-Rodriguez, Raynald Pineault

**Affiliations:** 1 Faculty of Nursing, Université de Montréal, Montreal, Quebec, Canada; 2 Department of Social and Preventive Medicine, Université de Montréal, Montreal, Quebec, Canada; 3 Centre de santé et de services sociaux de Bordeaux-Cartierville-Saint-Laurent, Quebec, Canada; 4 University Hospital of Navarra, University of Navarra, Pamplona, Spain; 5 Public Health Directorate, Montreal, Quebec, Canada

## Abstract

**Background:**

The new forms of organization of healthcare services entail the development of new clinical practices that are grounded in collaboration. Despite recent advances in research on the subject of collaboration, there is still a need for a better understanding of collaborative processes and for conceptual tools to help healthcare professionals develop collaboration amongst themselves in complex systems. This study draws on D'Amour's structuration model of collaboration to analyze healthcare facilities offering perinatal services in four health regions in the province of Quebec. The objectives are to: 1) validate the indicators of the structuration model of collaboration; 2) evaluate interprofessional and interorganizational collaboration in four health regions; and 3) propose a typology of collaboration

**Methods:**

A multiple-case research strategy was used. The cases were the healthcare facilities that offer perinatal services in four health regions in the province of Quebec (Canada). The data were collected through 33 semi-structured interviews with healthcare managers and professionals working in the four regions. Written material was also analyzed. The data were subjected to a "mixed" inductive-deductive analysis conducted in two main stages: an internal analysis of each case followed by a cross-sectional analysis of all the cases.

**Results:**

The collaboration indicators were shown to be valid, although some changes were made to three of them. Analysis of the data showed great variation in the level of collaboration between the cases and on each dimension. The results suggest a three-level typology of collaboration based on the ten indicators: active collaboration, developing collaboration and potential collaboration.

**Conclusion:**

The model and the typology make it possible to analyze collaboration and identify areas for improvement. Researchers can use the indicators to determine the intensity of collaboration and link it to clinical outcomes. Professionals and administrators can use the model to perform a diagnostic of collaboration and implement interventions to intensify it.

## Background

Current health policy in most Western countries calls for more effective delivery of accessible, continuous and comprehensive services. This phenomenon is related to a trend to new forms of healthcare organization, such as integrated care, health networks and program management–to all three of which collaboration is fundamental. These new forms of organizing services require not only the implementation of new structures, but also the development of new clinical practices based on collaboration. Health professionals are thus confronted with a demand for both interprofessional and interorganizational collaboration [1-3], implying a new division of clinical labour between professionals in different disciplines and between different types of primary-, secondary- and tertiary-care institutions. In these new forms of organization, responsibility for coordination is shifted to the care providers and must therefore become more explicit and transparent [4]. Health professionals must consequently shoulder more and more of the burden of the collaborative and coordinating activities required in clinical settings.

Although decision makers increasingly assert their interest in promoting a transition to interprofessional and interorganizational collaboration, effecting the shift is no easy matter [5]. The problems stem from a number of interactional, organizational and sociopolitical factors [6-10]. Though recent advances have been made in research on collaboration, professionals need conceptual tools to help them develop collaboration amongst themselves in complex systems. If we are to effect a true transition to more integrated forms of organization, we need to better understand collaborative processes.

Perinatal services, particularly short stays or "early obstetric discharge," are an especially sensitive issue for interprofessional and interorganizational collaboration. Early obstetric discharge has become general practice in most Western countries [11], and collaboration is one of the methods of ensuring accessibility and continuity of care in postnatal follow-up and in the prevention of such problems as the cessation of breastfeeding, hospitalization of the baby for jaundice [12] and parent-child-attachment issues.

### A model for understanding the structuration of collaboration

The frame of reference for this study is the structuration model of collaboration, which applies to interprofessional and interorganizational collaboration in healthcare organizations. The model can be used to analyze the ways in which increasingly complex and heterogeneous multi-level systems of actors collaborate. It was developed by D'Amour following a study of interprofessional collaboration in a primary-healthcare setting [13] and is based on the concept of collective action in organizational sociology, specifically in strategic analysis as developed by Crozier and Friedberg [14] and in Friedberg's organizational approach [15]. For these authors, organizational sociology is a type of reasoning for the analysis of political, social and economic processes. They see collective action arising locally as a product of the actions and behaviours of various partners.

Collaboration is the central problem in any collective undertaking [15,16]. It is based on the premise that professionals want to work together to provide better care. At the same time, though, they have their own interests and want to retain a degree of autonomy and independence; the main instrument for negotiating such autonomy is power. Drawing on the literature, we were able to develop a model that takes issues of structure into account but focuses on relationships between individuals and the interaction between the relationships and the organizational dimensions. The model has been tested in different collaborative settings: in teams [13,17], between organizations [18], and in integrated healthcare networks [19].

**Figure 1:**
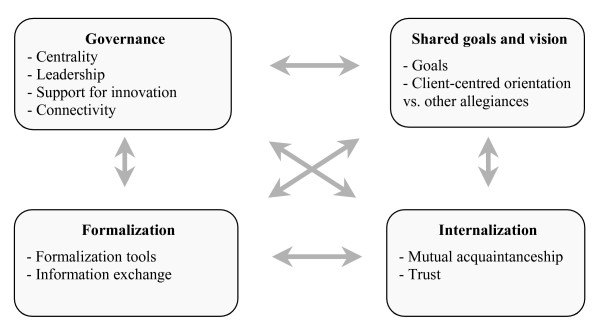
**The Four-Dimensional Model of Collaboration**. This figure shows the four dimensions of the model of collaboration and the ten indicators associated with these dimensions. The arrows indicate the interrelationships between the four dimensions and how they influence each other.

The model suggests that collective action can be analyzed in terms of four dimensions operationalized by 10 indicators (Figure 1). Two of the dimensions involve relationships between individuals and two involve the organizational setting (which influences collective action). As Figure 1 shows, the four dimensions are interrelated and influence each other. The relational dimensions are: 1) **Shared Goals and Vision**, which refers to the existence of common goals and their appropriation by the team, the recognition of divergent motives and multiple allegiances, and the diversity of definitions and expectations regarding collaboration; and 2) **Internalization**, which refers to an awareness by professionals of their interdependencies and of the importance of managing them, and which translates into a sense of belonging, knowledge of each other's values and discipline and mutual trust. One of the organizational dimensions is 3) **Formalization **(structuring clinical care), defined by Bodewes [20] as "the extent to which documented procedures that communicate desired outputs and behaviours exist and are being used" (p. 219). Formalization clarifies expectations and responsibilities. The other organizational dimension is 4) **Governance**, that is, the leadership functions that support collaboration. Governance gives direction to and supports professionals as they implement innovations related to interprofessional and interorganizational collaborative practices. Together, these four dimensions and the interaction between them capture the processes inherent in collaboration. They are subject to the influence of external and structural factors such as resources, financial constraints and policies. Though these factors are beyond the scope of this article, they must be taken into account as determinants of collaborative processes.

### Objectives of the study

The objectives of the study were threefold: 1) to validate the indicators of collaboration; 2) to evaluate interprofessional and interorganizational collaboration in perinatal care in four health regions in Quebec; and 3) to propose a typology of collaboration.

## Methods

A qualitative, descriptive, multiple-case research design was used [21]. The cases comprised the health facilities providing perinatal services in four health regions in the province of Quebec: two urban regions, one mixed urban-rural region and one rural region [18]. The regions were selected because they present different models of continuity of care, depending on whether responsibility for postnatal follow-up had or had not been transferred to community partners. Each region has one or two hospitals offering perinatal care, an average of six community health centres, several private medical clinics, and voluntary organizations providing postnatal support. In addition, each region has a regional agency, whose mission includes ensuring the integration and consistency of services between the various healthcare facilities on its territory. These facilities were expected to implement collaboration following introduction of the early discharge of newborns, defined as a postpartum stay of approximately 48 hours (2 days) for an uncomplicated vaginal delivery and approximately 96 hours (4 days) for an uncomplicated caesarean [3-5]. This practice requires linking hospital care with primary-care services.

The data were collected through 33 semi-structured interviews with healthcare managers and professionals working in the four regions (Table 1). An analysis of written material (coordination agreements, protocols, etc.) was also conducted. The sampling was purposeful; respondents were selected to represent management and the different types of professionals. An interview plan based on D'Amour's model of collaboration was developed to guide the interviews. All interviews were recorded on audio tape and transcribed in full.

**Table 1:** Number of individuals interviewed by region and type of establishment

**Type of establishment**	**Region A**	**Region B**	**Region C**	**Region D**
**Regional board**	1	2	-	1
**Hospital**	3	1	3	3
**CLSC**	3	5	4	3
**Community agency**	1	1	1	1

*The interview subjects are managers, physicians, nurses, and other healthcare professionals.

The data analysis combined two complementary strategies [22,23], deduction and induction. We thus based our analysis on the theoretical proposals of the analytical model of interprofessional collaboration but left room for new elements to emerge. Each stage in the analysis was conducted independently by three investigators who then compared their findings. The analysis comprised two main stages: an internal analysis of each case followed by a cross-sectional analysis of all the cases [24]. The first step involved three levels of analysis: condensation, organization and interpretation [23]. The units of meaning were thus coded in terms of the indicators of the model of collaboration or of the emerging themes. A history of collaboration and the factors influencing it was then written for each case and the cross-sectional analysis was then undertaken. The study was submitted for approval to the ethics committees of the University of Montreal and of the participating facilities, and all participants signed a consent form.

## Results

### The indicators of the model

In our earlier studies [13,18] we operationalized the four dimensions of the model, identifying ten indicators of the dimensions to evaluate processes of collaboration. There are four indicators for the relational dimensions and six for the organizational ones. One of the goals of this study was to validate these indicators. We therefore shifted back and forth continually between analyzing the data, validating the indicators and evaluating the level of collaboration, while leaving room for the emergence of new units of meaning. One of the first findings was that no new indicator emerged. The data allowed us to consolidate the definition of the ten indicators. Table 2 presents an operational definition of each indicator.

**Table 2:** Indicators of collaboration

	**Indicators**	**Description**
SHARED GOALS AND VISION	***Goals***	This indicator is related to professional values in the form of common goals, with particular reference to the consensual and comprehensive nature of the goals. Identifying and sharing common goals is an essential point of departure for a collaborative undertaking. The data suggest that the goal most likely to rally stakeholders is that of promoting patient-centred care. Providing a response to clients' needs thus becomes a central objective on which everyone can agree. The problem is that this goal entails a radical transformation of values and practices; its achievement would truly be an innovation.
	***Client-centred orientation vs. other allegiances***	There generally exists a complex structure of interests involving a variety of different types of allegiance: to the clientele, to the profession, to the organization, to private interests, etc. The result is thus an asymmetry of interests among partners or a partial convergence of interests. Mutual adjustments are required, making the need to negotiate all the more important. In some cases, negotiation is possible. In others, interests are left largely unexpressed, and there is no negotiating process. When shared goals are not negotiated, the risk is that private interests will emerge, resulting in opportunistic behaviour and a concomitant loss of focus on client-centred collaboration.

INTERNALIZATION	***Mutual acquaintanceship***	The data show that professionals must know each other personally and professionally if they are to develop a sense of belonging to a group and succeed in setting common objectives. Knowing each other personally means knowing each other's values and level of competence. Knowing each other professionally means knowing each other's disciplinary frame of reference, approach to care and scope of practice. The familiarization process occurs at social occasions, training activities and formal and informal information-exchange events. It is necessary to create the social conditions that will foster collaboration, particularly through social interaction.
	***Trust***	According to the professionals, collaboration is possible only when they have trust in each other's competencies and ability to assume responsibilities (that is, when goodwill exists). Trust reduces uncertainty. Professionals acknowledge that they do not know each other well, and so must constantly gauge risks and allow themselves to be placed in a vulnerable position. When there is too much uncertainty, the data show, health professionals hold on to responsibility for their clients as long as possible to avoid collaborating. Such actions run counter to the goal of constructing networks. Professionals use the results of collaboration to evaluate each other and build trust.

GOVERNANCE	***Centrality***	Centrality refers to the existence of clear and explicit direction that is meant to guide action, in this case, towards collaboration. The data reveal the importance of the involvement of some central authorities in providing clear direction and playing a strategic and political role to further the implementation of collaborative processes and structures. Senior managers can exert significant influence on interorganizational collaboration, particularly through agreements they reach with the managers of other facilities to make the collaboration official.
	***Leadership***	Local leadership is necessary for the development of interprofessional and interorganizational collaboration. Leadership may take a variety of forms and can be categorized as either emergent or as related to a position. With respect to collaboration, leadership can be exercised either by managers who have been mandated to do so or by professionals who take the initiative themselves. In the latter case, leadership is shared by the different partners and is subject to wide agreement. When leadership is related to a position, power should not be concentrated in the hands of a single partner; all partners must be able to have their opinions heard and to participate in decision making.
	***Support for innovation***	Because collaboration leads to new activities or because it involves dividing responsibilities differently between professionals and between institutions, it necessarily entails changes in clinical practices and in the sharing of responsibilities between partners. These changes represent real innovations that must be developed and implemented. Collaboration cannot take hold without a complementary learning process and without the organization involved drawing on internal or external expertise to support this learning process.
	***Connectivity***	Connectivity refers to the fact that individuals and organizations are interconnected, that there are places for discussion and for constructing bonds between them. Connectivity is the opposite of being cut off, isolated, separate. It solves coordination problems and makes it possible to make adjustments to practices. Connectivity allows for rapid and continuous adjustments in response to problems of coordination. It takes the form of information and feedback systems, committees, etc.

FORMALIZATION	***Formalization tools***	Formalization is an important means of clarifying the various partners' responsibilities and negotiating how responsibilities are shared. There are many types of formalized tools: interorganizational agreements, protocols, information systems, etc. For professionals, it is important to know what is expected of them and what they can expect of others. Earlier findings suggest that collaboration is influenced less by the degree of formalization than by the consensus that emerges around formalization mechanisms and the specific rules that are implemented.
	***Information exchange***	The exchange of information refers to the existence and appropriate use of an information infrastructure to allow for rapid and complete exchanges of information between professionals. The findings suggest that professionals use information systems to reduce uncertainty in their relationships with partners they do not know well. Feedback provides professionals with the information they need to follow up with patients as well as to evaluate their partners on the basis of the quality of the written exchanges and feedback. This is an important aspect of establishing relationships of trust.

Three of the indicators were modified or refined. The indicator "expertise" was thus changed to "support for innovation", for our data revealed that the changes entailed in introducing collaborative practices are comparable to those involved in implementing innovations. The data also allowed us to refine our definition of the indicators "centrality" (central leadership) and "local leadership". The central-leadership indicator, which essentially involved the existence of a strong central authority, was changed by the addition of a strategic role in promoting collaboration. The local-leadership indicator was improved by bringing to light different types of leadership: emergent versus position-related.

Another finding was the significant variation in the achievement of the indicators, which led to the establishment, for each indicator, of a continuum ranging from 1 to 3; 1 representing the minimum level of achievement of an indicator and 3 the maximum. The levels 1 to 3 for each indicator are explained in Table 3. Reference to the levels makes visual representations of collaboration possible.

**Table 3:** Indicators of collaboration according to the typology

**Indicators**	**Active Collaboration LEVEL 3**	**Developing Collaboration LEVEL 2**	**Potential or Latent Collaboration LEVEL 1**
**Goals**	Consensual, comprehensive goals	Some shared ad hoc goals	Conflicting goals or absence of shared goals
**Client-centred orientation vs. other allegiances**	Client-centred orientation	Professional or organizational interests drive orientations	Tendency to let private interests drive orientations
**Mutual acquaintanceship**	Frequent opportunities to meet, regular joint activities	Few opportunities to meet, few joint activities	No opportunities to meet, no joint activities
**Trust**	Grounded trust	Trust is conditional, is taking shape.	Lack of trust
**Centrality**	Strong and active central body that fosters consensus	Central body with an ill-defined role, ambiguous political and strategic role.	Absence of a central body, quasi-absence of a political role.
**Leadership**	Shared, consensual leadership	Unfocused, fragmented leadership that has little impact	Non-consensual, monopolistic leadership
**Support for innovation**	Expertise that fosters introduction of collaboration and innovation	Sporadic, fragmented expertise	Little or no expertise available to support collaboration and innovation
**Connectivity**	Many venues for discussion and participation	Ad hoc discussion venues related to specific issues	Quasi-absence of discussion venues
**Formalization tools**	Consensual agreements, jointly defined rules	Non-consensual agreements, do not reflect practices or are in the process of being negotiated or constructed	No agreement or agreement not respected, a source of conflict
**Information exchange**	Common infrastructure for collecting and exchanging information	Incomplete information-exchange infrastructure, does not meet needs or is used inappropriately	Relative absence of any common infrastructure or mechanism for collecting or exchanging information

### Presentation of the cases

Because two of the four cases were similar in many ways, it was decided to describe only three of them in this article. Each case is presented with a brief description of the partners (such as number of partner institutions and special features). The findings are presented in terms of the four dimensions and the ten indicators of the model for each case and reproduced in schematic form in a Kiviat graph, which makes it possible to visualize the shortfalls between the current situation (on a continuum from 1 to 3 depending on level of achievement) and optimal collaboration.

#### Case 1. Region A

##### Background of Region A

Eight partners – two hospitals and six CLSCs (community health centres) – provide postnatal services. Several private medical clinics are also involved. The regional board is actively involved in supporting collaboration between the partners. Two main characteristics emerge. Firstly, as compared to other Quebec regions, the population is largely socioeconomically disadvantaged. Secondly, the region is underfinanced as far as the health and social services workforce is concerned. The partners consequently realized that they had to pool their resources in order to achieve their health and social service objectives.

**Figure 2:**
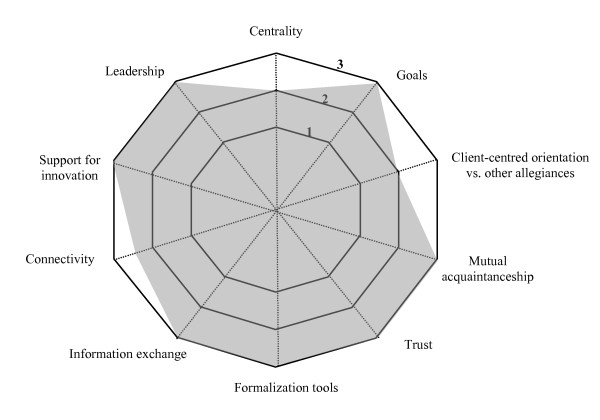
**Indicators of collaboration – Region A**. This Kiviat graph maps collaboration in Region A. A score of 1 to 3 is assigned to each of the 10 indicators depending on the level of achievement of the indicator in the region. The graph helps visualize the gap between optimal collaboration processes (Level 3) and the current situation.

On the basis of the indicators of the structuration model of collaboration, we see that, despite shortfalls, Region A was able to develop a significant level of collaboration. The Kiviat graph (Figure 2) provides a schematic view of collaboration in Region A.

In terms of the Shared Goals and Vision dimension, the participants' responses show that the partners from the hospital and the community health centres in Region A have succeeded in acquiring a common set of goals: notably the goal of increasing parental involvement in the pre- and postnatal period. This point is clearly expressed by one of the hospital health professionals:

*I think that at the outset there was a good agreement with the people. Then we got acquainted with each other. Ultimately, we share the same goal, so we pool all our knowledge .... Our common goal was our mothers, the spouses and the babies. (1: 155–159) *[Translation]

To attain their goal the partners developed joint projects, such as the introduction of a pregnancy logbook. The participants considered that professional and organizational interests must in no way take precedence over the quality of services to the clients, and they took steps to settle issues regarding such interests that might hamper pursuit of their goals. In Region A, the Shared Goals and Vision dimension is characterized by the existence of comprehensive common goals centred on client needs.

With respect to the dimension of Internalization, the data show that at the outset relations between the professionals from the various organizations were marked by mistrust and prejudice. For example, the hospital physicians displayed a lack of trust in the nurses from the community centres:

*It's not necessarily that they don't place any trust in the CLSC, but if you don't know what goes on in a CLSC, or you know virtually nothing, it's like putting your child into the care of someone you've never met.... If the child is yellow, will they be able to see it's yellow? Will they be able to do their work? It's a demanding approach because neither sector knows the other. (8:73) *[Translation]

The professionals have become acquainted with each other thanks, most notably, to the introduction of such methods as joint training for hospital and community personnel, visits to the different facilities and the establishment of working committees. These developments fostered the growth of trust between professionals from the different organizations, and a new division of responsibilities between hospital and CLSC personnel could consequently be implemented. As one of the professionals interviewed put it:

*But now the people from here know the CLSC people as well as they know us, and we even have skills development for care providers here onsite. All the perinatal care nurses from the CLSCs came for training on how to conduct a physical examination of the newborn and postpartum. The training period wasn't very long, half a day, but they saw the paediatrician who was there, and he showed them how to conduct good in-home observations, examine the baby and the mother and everything. (1:149) *[Translation]

In terms of the Governance dimension, the results show that Region A is characterized by a strong central authority that provides clear direction. Even so, as noted earlier, it could not bring all groups together, particularly the physicians from private clinics. Governance in this region is also characterized by strong, emerging, consensual local leadership. The leadership of one hospital nurse and one CLSC nurse helped collaboration emerge. In this region, the public health department provided the expertise to support the professionals in the introduction of new and innovative practices (training, program development, project funding, etc.), thus fostering strong, comprehensive involvement by the professionals from the different healthcare facilities. The partners also worked to improve connectivity and communications by setting up working committees and consultative forums where the professionals could bring their complaints so that the necessary adjustments can be made as issues arise and before the situation deteriorates:

*Before, we'd be told, "That's no good. It's not right. I have a complaint to make about you." Now, it's managed case by case. Instead of saying "The hospital isn't right," ... we have the file sent over and look at it. (1: 161–163) *[Translation]

The Formalization dimension of collaborative relationships in this region is based mainly on an agreement drawn up jointly by the partners. It is very detailed and sets out each partner's responsibilities and the procedures to follow in the event of conflict. The partners have generally respected the agreement. They thus supported the swift settlement of any problems that arose and made sure services could develop. In addition, the mechanisms for exchanging information between the partners have evolved over time and played an important role throughout the collaboration-development process to meet the objective of ensuring continuity of care.

On the whole, based on the indicators, the results reveal that Region A has put a significant level of collaboration into practice.

#### Case 2. Region B

##### Background of Region B

Eight healthcare establishments–seven CLSCs and one hospital–serve a large area comprising a diverse rural, industrial and urban population. There are several private medical clinics and community agencies. The regional board is not very involved. The introduction of early discharge occurred against a backdrop of major changes in both organizational structure and professional practice. The merger of some facilities, including two hospitals and two CLSCs, had serious consequences that continue to be reflected in difficulties in integrating the different institutional cultures. The CLSCs did not adapt the way they organized services to deal with their new responsibilities under the shift to ambulatory care, while the hospital put into place some services for infant safety after discharge.

**Figure 3:**
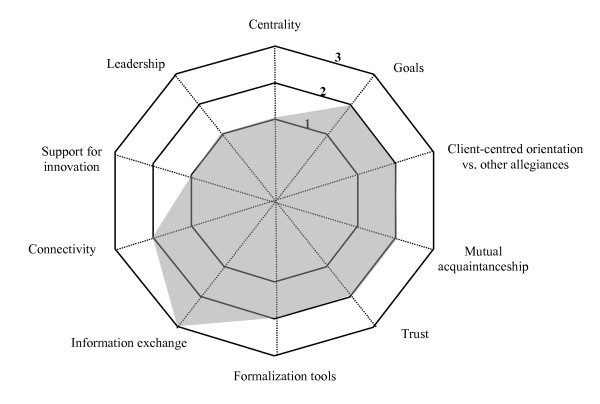
**Indicators of collaboration – Region B**. This Kiviat graphs lays out a schematic view of collaboration in Region B. A score of 1 to 3 is assigned to each of the 10 indicators depending on the level of achievement of the indicator in the region. The graph helps visualize the gap between optimal collaboration processes (Level 3) and the current situation.

Figure 3 presents the indicators of collaboration in Region B as they emerge from the analysis of the data.

With respect to the Shared Goals and Vision dimension, analysis of the data shows that the partners had only partial success in coming together around common goals. They developed a joint project on breastfeeding, but could not reach consensus on any other endeavours, such as in-home screening for jaundice or prenatal/postnatal continuity. The results show that some key actors in the region were more concerned with protecting their professional autonomy and the autonomy of their organization than with meeting the needs of the clients. One of the physicians said in reaction to early discharge:

*Six months ago, the paediatricians created a jaundice protocol that was stricter than before. Some doctors say, "I'll take it and keep it, then we'll take the system apart to show them the protocol makes no sense." (8:213–227) *[Translation]

Regarding the Internalization dimension, the healthcare professionals were unable to internalize the fact that they are a team. They have few opportunities to meet and socialize; few joint events or training activities have been arranged. Their weak sense of belonging to the team seems to be associated with a feeling of trust between hospital and CLSC personnel that is conditional and fragile. The partners have to demonstrate their competence to each other. However, the results show the level of trust is rising and has led to a more optimal division of responsibilities:

*There was a problem of trust. Even the hospital nurses and the CLSC nurses didn't trust in each other. They questioned each other's competence in caring for the mother or baby. We realize now that the establishments know each other better.... It's still far from perfect, but in terms of harmonizing perinatal care, say, it's a lot better than it was. (9: 25,83) *[Translation]

*It's recent, not something that's been around for many years, us being able to sit down and say who does what, us being able to trust in each other. I think that knowing each other means we can place our trust in each other because the fact remains that the professionalism is there and the quality of care is as important to us as it is to them.... You don't get to know people overnight. Now we're more in the way of being collaborators. (7: 83)*. [Translation]

The dimension of Governance in the region is characterized by weak centrality. The central authority (regional board) restricts its role to arbitrating conflicts between the hospital and the CLSCs. It has not been able to give direction or influence action. Nor has it created the impression that it has the expertise to promote innovation in collaborative practices and thus provided the support needed to develop collaboration. Leadership is, as it were, preordained and monopolized by the hospital in a non-consensual fashion. The hospital physicians' position has been dominant.

With regard to the dimension of Formalization, an agreement was signed by the CLSCs and the hospital which defines the goals, the services offered and the method for exchanging information. However, the agreement does not specify how clinical responsibilities are to be divided between facilities. The respondents claim to be satisfied with the mechanisms for exchanging information.

To sum up, the partners in this region have developed an active collaboration in the sense that it is evolving but remains incomplete.

#### Case 3. Region C

##### Background of Region C

Six partners provide perinatal services: four CLSCS, one hospital and one ambulatory centre. The creation of the ambulatory centre caused dissension over the division of responsibilities between it and the CLSCs. Home visits by ambulatory-services professionals encroach on CLSC responsibilities. The distribution of resources between these establishments is also a contentious issue in the region.  The clientele–young, middle-class families–is relatively homogeneous from one CLSC territory to the next. There is a great deal of movement between regions, and consequently the hospital and the ambulatory centre have entered into partnerships with other health regions.

**Figure 4:**
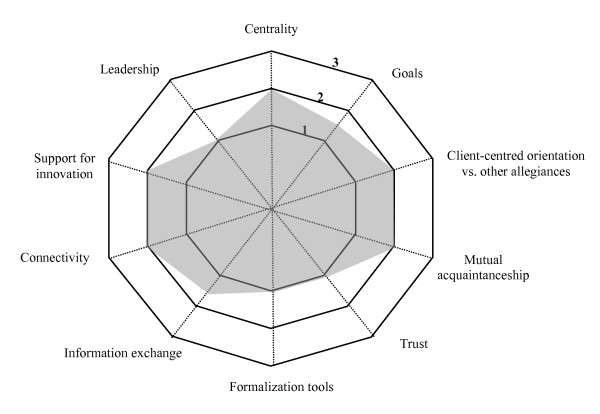
**Indicators of collaboration – Region C**. This Kiviat graphs lays out a schematic view of collaboration in Region C. A score of 1 to 3 is assigned to each of the 10 indicators depending on the level of achievement of the indicator in the region. The graph helps visualize the gap between optimal collaboration processes (Level 3) and the current situation.

Figure 4 presents the collaboration indicators for Region C. The disparity between the current situation and optimal collaboration is quite wide.

With respect to the Shared Goals and Vision dimension, analysis of the data shows that the healthcare professionals did not manage to consult with each other to set shared goals for the CLSCs, the hospital and the ambulatory centre. Professional, personal and organizational interests loom large and guide the professionals' actions. Indeed, they do not often mention responding to the clientele as an action strategy.

On the Internalization dimension, relations between personnel from the different establishments in the region are characterized by a lack of trust and the absence of opportunities to meet. Some professionals from the hospital and the ambulatory centre question the competence of the CLSC nurses and leave them little autonomy in following clients. Similarly, an attitude of mistrust of some hospital and ambulatory-services professionals developed in the CLSCs. The mutual lack of trust is so intense that even attempts at rapprochement, notably during training activities, have failed. As one CLSC nurse put it:

*It was hard for us to get our expertise recognized. If I see a baby at home, I observe things, and, according to my protocol, I have to refer it to the hospital, and we often came up against it. The baby got to the hospital, and they started all over again, doing what we had already done, like they were validating us. (2:253) *[Translation]

In relation to Governance, the data show that the central authority plays an ambiguous role. The regional agency's structures and responsibilities are not clearly established. According to many of the professionals interviewed, the central board must become more involved to direct the implementation of a collective approach by all the health facilities. The board provided expertise in a sporadic, fragmentary fashion. The medical establishment, which enjoys great influence over regional decision making, monopolized leadership on perinatal care in the region; as the following citation indicates, the CLSC actors felt excluded from decision making. Any consultation is restricted to specific cases.

*It's the four CLSCs against the hospital. No, they're not on an equal footing. Clearly, the hospital's medical power is very, very, very considerable, so there is a significant difference betwen the power we can have as a CLSC as opposed to what the hospital can have. (3:69) *[Translation]

Analysis of the Formalization dimension shows that although guidelines on the division of responsibilities were drawn up in Region C, they were non-consensual and conflictual. The guidelines took the form of intervention protocols that some partners, particularly the CLSCs, disagreed with. Hospital physicians dictate the rules unilaterally, leading the CLSCs to adopt strategies of protest and opposition. The channels for exchanging information also pose a problem for CLSC personnel.

In this region, then, there is little collaboration, and relations between the partners are quite conflictual.

Taking all three cases together, a great deal of variation is displayed in terms of the level of collaboration, providing a basis for fulfilling the third objective of this study: proposing a typology of collaboration.

### The typology of collaboration

A typology is derived from the analysis of several elements that are considered simultaneously in order to arrive at a given type of classification [25]. The typology we propose takes into account the degree of collaboration as shown by the ten indicators of the four dimensions of the model of collaboration.

The empirical data suggest a threefold typology: active collaboration, developing collaboration and potential collaboration.

▪ *Active collaboration *is collaboration of the highest level. The partners have successfully established stable collaboration that is sustained despite uncertainties in and shocks to the healthcare system. The partners have adopted common, consensual goals, developed a sense of belonging and mutual trust and reached consensus on mechanisms and rules of governance. As a result, professional practices should be transformed on the basis of a new consensual division of interprofessional and interorganizational responsibilities and the introduction of innovative practices. This type of collaboration is represented by Region A (Figure 2).

▪ *Developing collaboration *is collaboration that has not taken root in the cultures of the partner organizations and may still be subject to re-evaluation on the basis of internal or environmental factors. Goals, relationships between partners, governance mechanisms, and formalization are the subject of a negotiating process that has not yet produced a consensus. The negotiations may be partial or a source of conflict, but they are nevertheless open, ongoing and accessible. This type of collaboration results in a tentative division of responsibilities between professionals and institutions; in timid transformations of professional practices; and in services that are less efficient than they might be. In developing collaboration, we have observed that although change takes longer, progress is clearly being made. Region B exemplifies this type of collaboration (Figure 3).

▪ *Potential collaboration *refers to collaboration that does not yet exist or has been blocked by conflicts that are so serious that the system cannot move forward and satisfactory forms of collaboration cannot be implemented. When potential collaboration is characterized by significant opposing forces, either negotiations do not take place or they are constantly breaking down. It is therefore hard to introduce the new professional practices that the network needs, for innovation is difficult in an environment beset by a whole series of conflicts. Services may suffer from a loss of accessibility and continuity. Only by resolving the conflicts can collaboration be implemented. Region C represents this type of collaboration (Figure 4).

## Discussion

The three objectives of the study were to: 1) validate the collaboration indicators; 2) evaluate interprofessional and interorganizational collaboration in three health regions in Quebec; and 3) propose a typology of collaboration.

The analysis of collaboration we have presented is based on a model comprising four dimensions and operationalized with ten indicators that are evaluated simultaneously to provide a comprehensive view of the phenomenon. The collaboration indicators were shown to be valid, though some changes were made, chiefly to the support-for-innovation indicator. This change shows how important it is not to underestimate the implications of the novelty of the interprofessional approach and that resources thus have to be allocated to support it [26,27]. Moreover, it is important to recognize the influence of central and local leadership on collaboration [1,28]. High-level leaders have great credibility and so have a strategic role, both internally and externally, in promoting collaboration. Meanwhile, local leaders are the best placed to ensure that organizational barriers to collaboration are eliminated.

The case analysis helped us understand some of the processes involved in building collaboration. The four dimensions seem to reveal enough of these processes that we can identify aspects that partners should work on in this endeavour. The evaluation reveals a great deal of variance in the level of collaboration in the three cases and on each of the dimensions. For example, on the dimension of Shared Goals and Vision, the partners managed to acquire common goals in only one case (Region A); fruitful collaboration was developed based on the partners' desire to focus their activities on the needs of the clients. Many authors underscore the importance of professionals focussing on clients' needs, preferences and expectations [29,30]. In the other two cases (Regions B and C), however, the goals of the healthcare professionals were only partially shared. As we see, professional interests are hard to subordinate to the interests of the clientele.

The dimension of Internalization, developing a relationship of trust, was a challenge in every case. Only in Region A was it attained. The transcript of the interviews shows clearly how fundamental trust is, a point well documented in the literature [31]. In the study, trust is based primarily on a perception of the competence of others. Without trust, there seems to be no possibility of developing a collaborative approach [32]. In the area of perinatal care, the fact that newborns are so fragile makes the need for trust in one's partners all the more important.

The dimension of Governance, the literature stresses, is crucial [33]. Its significance in this study can be gauged by the fact that in Regions B and C, where it is lacking, the partners have not managed to develop collaborative practices. In neither case was guidance or direction given to foster collaboration. Nor was there strong local leadership to encourage and facilitate collaboration by providing venues for connectivity and establishing a climate of safety and participation [34]-.

The Formalization dimension proved necessary for clarifying the division of responsibilities between the different establishments and ensuring that issues are dealt with quickly. However, formalization has been implemented in only one case. Even when agreements exist, they are not necessarily consensual, and they create conflict.

These results show that it is difficult to identify a single dimension that takes precedence over all the others; the organizational and relational dimensions of collaboration should thus be considered simultaneously [35,36].

We have proposed a typology of collaboration based on the ten indicators as a means of taking all the dimensions into account. The literature on collaboration often cites two other typologies. Ivey et al. [37] suggest that interprofessional collaboration exists along a continuum of intensity ranging from parallel practices to interdisciplinary teams. Alter and Hage [38] deal with forms of interorganizational collaboration (sequential, reciprocal and collective). Though both typologies are very useful, neither provides any explicit indicators of collaboration. We have therefore proposed a typology based on explicit indicators that can be used to perform a diagnostic of collaboration. The significantly different levels of collaboration observed in these cases demonstrate that there are indeed different forms or stages of collaboration and suggest that it evolves along different paths.

### Limits of the study

The findings provide indicators for evaluating collaboration and are thus of some importance. The study does have its limitations, though. Firstly, the model cannot capture all the finer points of a concept as complex as collaboration. Nor does it capture all the factors that can influence collaboration; its main objective is to examine processes of collaboration. The model thus takes into account certain structural, interactional and professional factors, albeit not exhaustively. Schmitt [39] stresses that collaboration is a multidimensional phenomenon: it may be conceptualized as a structure, as a process or as intermediate outcomes.

Another limitation that should be stressed is that the model focuses on collaboration between health professionals. What place should clients and families be accorded in such a model? The model does make it possible to gauge the extent to which professionals are or are not focussed on the interests of patients. An important subject for research would be to go further to understand the interests of patients and families and the place they would like to be accorded in interprofessional collaboration. There is an extensive literature on the necessity of patient participation in collaboration, but no determination of how to attain it [40,41]. Use of this model might advance research on bringing about collaboration between patients and healthcare professionals to construct collective action.

The generalizability of the findings on the validation of the indicators may be limited by the fact that the study bears on the collaboration of professionals in different organizations. Do the results apply to collaboration within the same organization or to collaboration between other partners, for example, to intersectorial collaboration? Would the same indicators apply? Further research is needed to elucidate these points. The same questions arise in terms of the generalizability of the model to other cultures. For all these reasons, the precise description of the context that we have provided will help readers assess the applicability of the findings to other settings.

For each indicator, a continuum ranging from 1 to 3 was developed, and the operational indicators are helpful for visualizing collaboration. However, more validation is needed of the link made between the 10 indicators and the classification into types of collaboration.

## Conclusion

Collaboration is an integral part of everyday life, and under certain conditions it can be transformed into collective action. The model we have presented recognizes the complexity of the phenomenon. A diagnostic of collaboration should take ten different indicators into account. This approach is similar to propositions in the literature on team-building, which suggest intervention at several levels of an organization [42] and in several areas [8,34]. One limitation of the model is that it cannot capture all the finer points of a concept as complex as collaboration. Nonetheless, the model makes it possible to analyze collaboration comprehensively enough that shortfalls between effective and optimal collaboration can be identified and areas for improvement highlighted.

Researchers can use the indicators to determine the intensity of collaboration and link it to other variables, including clinical outcomes. The model can be used by professionals and administrators to perform a diagnostic of collaboration and implement interventions in order to intensify it. The typology, which can incorporate several indicators, proves most useful when implementing collaboration interventions, which, by their very nature, involve several dimensions.

## Competing interests

The authors declare that they have no competing interests.

## Authors' contributions

DD developed the collaboration model and carried out the collaboration study. LG developed the study design. JFL participated in the design of the study and the data analysis. LSMR participated in the data analysis and helped draft the manuscript. RP participated in the data analysis and helped draft the manuscript. All authors read and approved the final manuscript.

## Pre-publication history

The pre-publication history for this paper can be accessed here:


